# Non-osteopenic Bone Pathology After Allo-hematopoietic Stem Cell Transplantation in Patients with Inborn Errors of Immunity

**DOI:** 10.1007/s10875-023-01465-z

**Published:** 2023-03-17

**Authors:** Zainab M. Golwala, Nikita Gireesh Bhat, Jinhua Xu-Bayford, Tanja Stankova, Stuart Adams, Emma C. Morris, Waseem Qasim, Claire Booth, Austen Worth, Maaike A. Kusters, Reem Elfeky

**Affiliations:** 1 Department of Immunology, Great Ormond Street Hospital for Children NHS Foundation Trust, Great Ormond Street, London, WC1N 3JH UK; 2 UCL Great Ormond Street Institute of Child Health, London, UK; 3 SIHMDS-Haematology, Great Ormond Street Hospital for Children NHS Foundation Trust, London, UK; 4 Department of Immunology, Institute of Immunity and Transplantation, University College London, London, UK

**Keywords:** Bone pathology, HSCT, Inborn errors of immunity, Skeletal dysplasia, Bone marrow transplantation, Osteochondroma, WAS, Osteopenia, Long term follow-up

## Abstract

**Purpose:**

There is a lack of data on post-HSCT non-osteopenic bone pathology specifically for children with inborn errors of immunity (IEI). We collected data on non-osteopenic bone pathology in children with IEI post-HSCT over two decades in a large tertiary pediatric immunology center.

**Methods:**

Descriptive study with data analysis of bone pathology in allo-HSCT for IEI was performed between 1/1/2000 to 31/12/2018 including patients alive at follow-up to July 2022. Records were analyzed for bone pathology and risk factors. Exclusion criteria included isolated reduced bone density, fractures, and skeletal anomalies due to underlying IEI and short stature without other bone pathology. Bone pathologies were divided into 5 categories: bone tumors; skeletal dysplasia; avascular necrosis; evolving bone deformities; slipped upper femoral epiphysis.

**Results:**

A total of 429 children received HSCT between 2000 and 2018; 340 are alive at last assessment. Non-osteopenic bone pathology was observed post-HSCT in 9.4% of patients (32/340, mean 7.8 years post-HSCT). Eleven patients (34%) had > 1 category of bone pathology. Seventeen patients (17/32; 53%) presented with bilateral bone pathology. The majority of patients received treosulfan-based conditioning (26/32; 81.2%). Totally, 65.6% (21/32) of patients had a history of prolonged steroid use (> 6 months). Pain was the presenting symptom in 66% of patients, and surgical intervention was required in 43.7%. The highest incidence of bone pathologies was seen in Wiskott-Aldrich syndrome (WAS) (*n* = 8/34; 23.5%) followed by hemophagocytic lymphohistiocytosis patients (*n* = 3/16; 18.8%).

**Conclusion:**

Non-osteopenic bone pathology in long-term survivors of allo-HSCT for IEI is not rare. Most patients did not present with complaints until at least 5 years post-HSCT highlighting the need for ongoing bone health assessment for patients with IEI. Children presenting with stunted growth and bone pathology post-HSCT should undergo skeletal survey to rule out development of post-HSCT skeletal dysplasia. Increased rates and complexity of bone pathology were seen amongst patients with Wiskott-Aldrich syndrome.

**Supplementary Information:**

The online version contains supplementary material available at 10.1007/s10875-023-01465-z.

## Introduction

Allogenic hematopoietic stem cell transplantation (Allo-HSCT) is the primary curative treatment for many inborn errors of immunity (IEI), with increasing numbers of patients treated over the last two decades. Improved quality of care pre- and post-HSCT has led to an improved overall survival rate for patients treated with HSCT, with improved management of early post-HSCT complications and long-term surveillance for earlier detection and management of late effects [1, 2]. Late effects such as pulmonary and cardiac complications are well described [3, 4] but late effects resulting in bone abnormalities are not well described, particularly in patients with IEI. Osteopenia has been reported among 25–50% of pediatric and adult recipients of stem cell transplant for malignant disease [5–7]. Multiple factors contribute to osteopenia and osteoporosis in this cohort including previous chemotherapy/radiotherapy for management of the underlying disease, conditioning chemotherapy, and polypharmacy, in particular the use of steroids for management of graft versus host disease (GvHD) or autoimmunity [8, 9].

Osteochondroma has been reported post-HSCT in patients with underlying malignant disorders and was noted in 5–20% of patients who received radiotherapy for malignancy such as neuroblastoma or in the context of total body irradiation (TBI)-based conditioning for HSCT [10–12]. Exposure to radiotherapy was considered a significant risk factor for the development of this benign bone tumor [10, 13, 14].

However, there are scarce data on non-osteopenic bone pathology post-HSCT in patients with IEI. Botto et al. recently described a pattern of spondyloepimetaphyseal skeletal dysplasia in 7 children who received HSCT for IEI which included 4 hemophagocytic lymphohistiocytosis (HLH) (2 perforin deficiency, 1 Munc-13, 1 secondary to Leishmaniasis) and 2 recombination activating gene-1(RAG-1) severe combined immunodeficiency (SCID) patients [15].

Great Ormond Street Hospital (GOSH) is one of two supra-regional UK centers delivering HSCT for children with IEI. A long-term follow-up program for children post HSCT is in place, allowing for systematic monitoring and data collection over time. Here, we report all non-osteopenic bone pathology in children with IEI who underwent HSCT in our center between 2000 and 2018.

## Methods

### Patient Characteristics

Records for patients with IEI who underwent allo-HSCT at GOSH between 1/1/2000 to 31/12/2018 were analyzed. Inclusion criteria included patients who were alive at last follow-up (up to 1st of July 2022). Exclusion criteria included patients with reduced bone density, fractures, scoliosis, and short stature without any other underlying non-osteopenic bone pathology or non-osteopenic bone pathology related to underlying IEI. Patient records were checked for non-osteopenic bone pathology and potential risk factors: age, gender, underlying IEI, transplant, intensity of conditioning regimen, stem cell source, onset and duration of steroid therapy, onset of hormonal replacement therapy (HRT) including gonadal hormones, gonadotropin-releasing hormone analogues (GnRHa) and growth hormone (GH), body mass index (BMI) in centiles at onset of bone disease, and donor engraftment at time of onset of bone disease and last follow-up. BMI > 91% was considered overweight. Written informed consent was obtained from all parents.

### Statistical Analysis

This is a descriptive analysis of patients who developed non-osteopenic bone pathology. Statistical analysis was performed using SPSS, version 24. Descriptive analyses were performed using median, mean, minimum, and maximum.

## Results

### Patients and Transplant Characteristics of the Studied Cohort

Patients (429) with IEI underwent 557 HSCT procedures (including CD34 top-up and donor lymphocyte infusion(s) (DLI)). Patients (79%; 340/429) were alive at last follow-up. Main IEI indications for HSCT in our center were SCID (*n* = 153; 45%), chronic granulomatous disease (CGD) (*n* = 42; 12%), and Wiskott-Aldrich syndrome (WAS) (*n* = 34; 10%). Most patients received either a treosulfan (Treo-) or busulfan (Bu-) based conditioning (295/340). Melphalan (Mel-) based conditioning was used in 13% of our studied cohort (45/340).

### Patient Demographics, Disease Category, and HSCT Characteristics of Patients with Non-osteopenic Bone Disease

In the overall cohort, 9.4% of the patients (32/340) developed non-osteopenic bone pathology. Patient demographics, disease category, and HSCT characteristics are described in Supplementary table 1. The mean age at first transplant was 2.74 years (range: 0.39–13.8 years), and there was a male predominance (71.8% (23/32) of the patients). Highest rates of bone pathology were observed in patients with WAS followed by HLH. Patients (10%) underwent HSCT for WAS of which 23.5% (8/34) developed bone pathology, 4.7% of patients underwent HSCT for HLH of which 18.8% (3/16) developed bone pathology suggesting a disproportionate incidence. On the contrary, the rates of bone pathology were less than 10% among the two most frequent diagnoses: SCID and CGD. Furthermore, no bone pathology was recorded among patients with cluster of differentiation 40 ligand (CD40L) deficiency, dedicator of cytokine 8 d (DOCK8) deficiency, immune dysregulation, polyendocrinopathy, enteropathy X-linked (IPEX), or activated PI3K delta syndrome (APDS). Table 1 shows a breakdown of IEI diagnoses for the overall cohort and rates of bone pathology in each IEI.

**Table 1:** Patient distribution with underlying IEI and bone disease post HSCT

Diagnosis	Number of patients who received HSCT (% of total transplants) (*n* = 340)	Number of patients who developed bone disease (% of bone disease) (*n* = 32)
SCID	153 (45)	13 (8.4)
CGD	42 (12.3)	1 (2.3)
WAS	34 (10)	8 (23.5)
CID	24 (7)	2 (8.3)
Immunodeficiency with intestinal disorders	19 (5.5)	2 (10.5)
XLP (excluding HLH as presentation)	12 (3.5)	1 (8.3)
HLH (including XIAP and XLP)	16 (4.7)	3 (18.8)
LAD/other neutrophil defect	11 (3.2)	2 (18)
XIAP (excluding HLH as presentation)	2 (0.5)	0
CD40 ligand deficiency	12 (3.5)	0
DOCK8 deficiency	8 (2.3)	0
IPEX	4 (1.1)	0
APDS	3 (0.8)	0

*APDS* activated PI3K delta syndrome; *CGD* chronic granulomatous disease; *CID* combine immunodeficiency; *CD40* cluster of differentiation 40; *DOCK8* dedicator of cytokine 8; *HLH* hemophagocytic lymphohistocytosis; *IPEX* immune dysregulation, polyendocrinopathy, enteropathy X-linked; *LAD* leucocyte adhesion defect; *SCID* severe combined immunodeficiency; *WAS* Wiskott-Aldrich syndrome; *XIAP* X-chromosome-linked inhibitor of apoptosis; *XLP*: X-linked lymphoproliferative disease

Three patients received either a second HSCT (*n* = 2) or DLI (*n* = 1) for management of graft loss or mixed chimerism. Conditioning regimen included Treo- or Bu-based conditioning in 25 and 2 cases, respectively. Seven patients received a fludarabine/melphalan (Flu/Mel) conditioning regimen. Only one patient received localized radiotherapy ahead of HSCT for treatment of malignancy (P16). Peripheral blood stem cells (PBSCs) were the main stem cell source in 19 transplants followed by bone marrow (BM) in 10 patients and cord blood in 5 patients. Seventeen (53.1%) of patients received a 10/10 matched unrelated donor (MUD), 1 received a matched family donor (MFD), 14 (43.7%) received 7–9/10 mismatched unrelated donor (MMUD) transplants, and amongst the 2 haploidentical transplants, one received a haploidentical TCR alpha/beta depleted HSCT followed by genetically modified T cell add-back as part of a clinical trial (NCT02065869), while the other received a OKT3 conditioning. Grade I/II acute GvHD (aGvHD) occurred in 50% of patients (16/32), grade III/IV in 5 patients, and 28% of patients (9/32) developed chronic GvHD (cGvHD). Patients (81%; 26/32) received steroid therapy, with 21 (66%) patients receiving prolonged steroid therapy for > 6 months (cumulative pre- and post-HSCT steroid exposure). Twelve patients (37.5%) were overweight (BMI > 91%), and short stature was present in 13 patients (40%) at the time of diagnosis of bone disease. Fourteen patients (43%) had gonadal dysfunction, of which 5 patients received hormonal therapy pre-bone pathology.

### Characteristics of Non-osteopenic Bone Pathologies

**Fig. 1:**
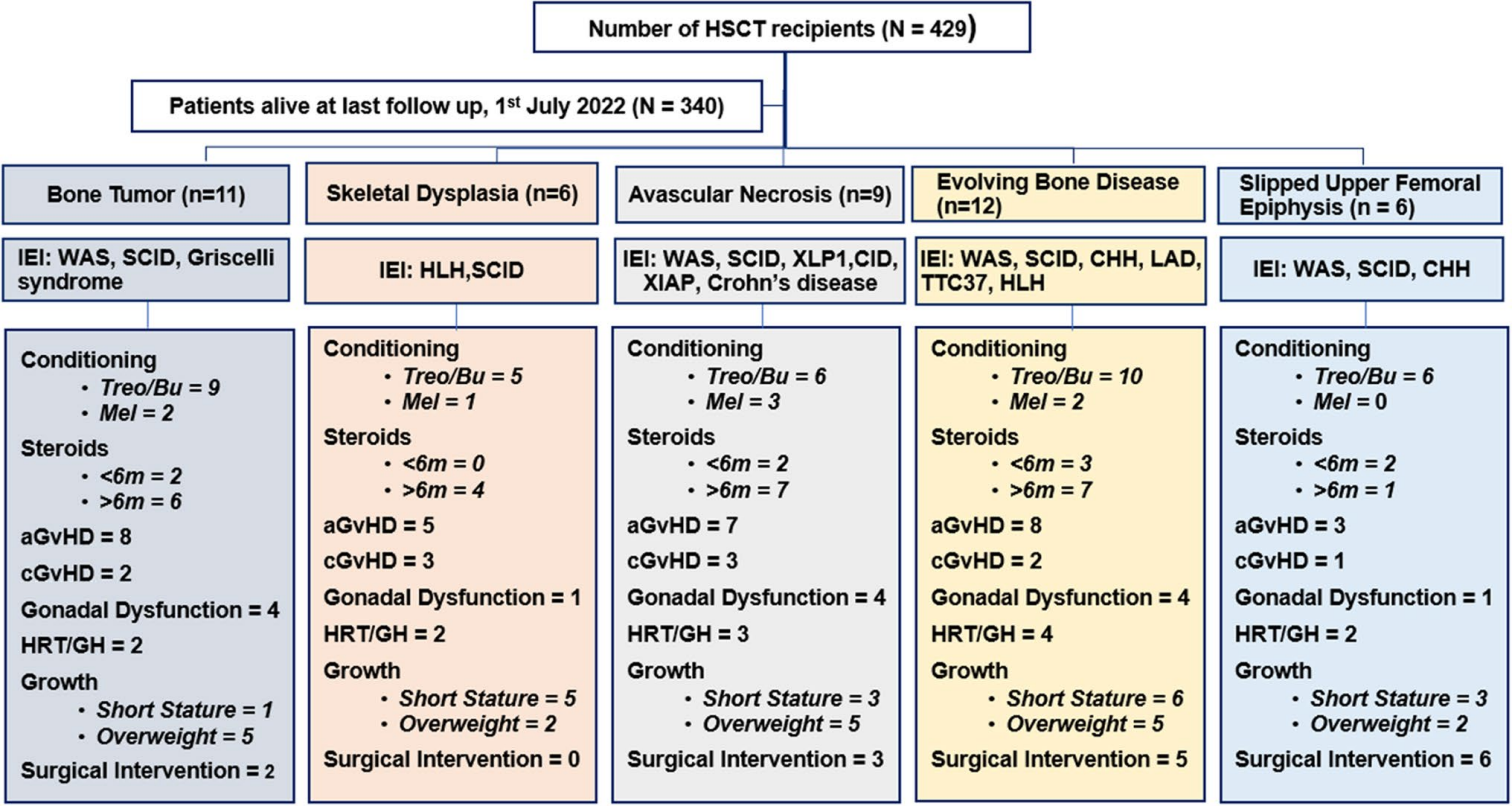
Flowchart for patients who developed bone diseases. Abbreviations: Treo/Bu, treosulfan or busulfan-based conditioning; aGvHD, acute graft vs host disease; cGvHD, chronic graft vs host disease; GH, growth hormone; HRT, hormone replacement therapy; Mel, melphalan-based conditioning; IEI, inborn errors of immunity; WAS, Wiskott-Aldrich syndrome; SCID, severe combined immunodeficiency; HLH, hemophagocytosis lymphohistocytosis; XIAP, X-chromosome-linked inhibitor of apoptosis; CHH, cartilage hair hypoplasia; XLP1, X-linked lymphoproliferative disease; CID, combined immunodeficiency; TTC37, trico-hepatoenteric syndrome

Bone pathologies were divided into 5 main categories; bone tumor (34%; *n* = 11), skeletal dysplasia (18.75%; *n* = 6), avascular necrosis (AVN) (28%; *n* = 9), evolving bone pathology (37.5%; *n* = 12), and slipped upper femoral epiphysis (SUFE) (18.75%; *n* = 6) (Fig. 1). One patient (P27) was previously reported by Botto et al. (15).

Patients (34%; 11/32) had more than one bone pathology. Mean time to developing first bone pathology was 7.8 years post-transplant (range: 2 to 16.2), with a mean follow-up of 10.6 years (range: 4.6–15). Twenty-one patients (65.6%) complained of pain ahead of diagnosis. Table 2 shows detailed description of patients with non-osteopenic bone pathology.

**Table 2:** Detailed description of patients with non-osteopenic bone pathology

ID	Type of BD	Pain/onset (years)/functional issues	Time of onset of BD post-HSCT∞ (years)	aGvHD	cGvHD	Steroid therapy/duration (months)	HRT/GH therapy (pre- vs post-onset of BD)	Others/need for surgery	BMI centile (percentage)	Chimerism at time of onset of bone disease	Chimerism at last follow-up/time of last follow-up (years)
P1	Dysplasia of both the tibia	Yes/12.25/abnormal gait	12.7	Grade IV-skin/gut/Liver	Chronic extensive gut	Yes 6–12 months	No	Gonadal dysfunction Short stature	96.9	WB 100%	WB 100% 14.6
P2	AVN of right femoral head	Yes/NA/NA	5.7	Grade I skin	Chronic extensive skin/liver/joint	Yes 24 months	No	Gonadal dysfunction Short stature	NA	WB 100%	WB 100% 6.1
P3	Frieberg disease of left second toe MTP (AVN)	NA	15	None	None	Yes > 12 months	Yes (pre)	Gonadal dysfunction	15.2	WB 100%	WB: 75% T:100% M: 71% 16.2
P4	AVN of right femoral head	Yes/2.3/mobility issues	2.5	Grade II skin	Extensive cGvHD/AI	Yes > 24 months	No	Gonadal dysfunction Surgical intervention	NA	WB 95% T:95% M:95% B:95%	WB:94% T:95% M:93% B:98% 4.8
P5	left fibular osteochondroma Scoliosis	None/11.14/no Pain with scoliosis	11.1 12.5	Grade II skin	None	Yes 3 weeks	NA	Gonadal dysfunction Surgical intervention	95.6	WB:100%	WB:100% 14.6
P6	Left SUFE Bilateral Knee genu valgum deformity	Pain/Not recorded/limp	11.07 12.6	None	None	Yes 3–4 months	None	Both had surgical intervention	76.8	WB:13% T:94% M:5%	WB:13% T:94% M:5% 14.3
P7	Bilateral exostosis of medial side of both knees	Pain/8.25/abnormal gait	11.18	None	Limited	No	None	None	41.3	WB:97%	WB:95% T:97% M:96% B:93% 12.5
P8	Right sided genu valgum	No/NA/abnormal gait	10.7	None	None	Yes 2.5 months	None	Gonadal dysfunction Short stature Surgical intervention	16.7	WB:100%	WB:100% 14.9
P9	Bilateral SUFE	Pain/8.2/abnormal gait with leg length discrepancy	9.31	None	None	No	Yes (post)	Delayed puberty Surgical intervention	99.6	WB:34% T:100% M:0% B:5%	WB:11% T:100% M:0% B:15% 14.5
P10	Bilateral genu valgum and foot deformities	Yes/not sure/no	8.9	None	None	Yes > 24 months	GH (pre)	Short stature on GH	89.6	WB:100%	WB:100% 10.5
P11	Bilateral genu valgum	Yes/6.02/abnormal gait with leg length discrepancy	8.7	Grade I skin	Extensive skin/joint	Yes > 12 months	No	None Surgical intervention	17.3	WB:79% T:80% M:84%	WB:62% T:60% M:61% 14.4
P12	Bilateral genu valgum	No/NA/knock knees	8.69	Grade III-skin/Gut	Extensive gut	Yes 10 months	No	Gonadal dysfunction Short stature Surgical intervention (X3)	94	WB:34% T:100% M:25% B:34%	WB:41% T:80% M:20% B:51% 12.6
P13	AVN of femur bilateral Osteochondroma right distal femur and left distal fibula Bilateral mild-moderate genu valgum	Yes/6.6/flexion deformity of the knee	6.68 10.65 10.65	Grade I skin	None	Yes 1 month	Yes (post)	Gonadal dysfunction	99.2	WB:100%	WB:100% 13.03
P14	Right Genu valgum deformity Exostosis of proximal right tibia	Yes/5.92/NA	8 10	Grade 1 skin	None	Yes > 12 months	Yes (post)	Gonadal dysfunction Surgery for genu valgum (X2)	99.9	WB:42% T:100% M:40% B:67%	WB:48% T:92% M:48% 13.15
P15	Left tibial exostosis Bilateral SUFE	No/NA/NA Yes/NA/NA	7 8.6	Grade III liver	Extensive-Kidney (nephrotic syndrome)	Yes > 24 months	No	Short stature Surgical intervention for SUFE	80.4	WB:100%	WB:100% 13.9
P16	Recurrent fractures- Genetics confirmed Osteogenesis imperfecta Osteosarcoma	Yes/2/limp Yes/3/limp	2 3	Grade II-skin and gut	None	None	No	None Surgical intervention	< 5% 10.8	WB 100%	WB 100% 5.6
P17	Bilateral SUFE Left genu valgum	Yes/6.78/limp Yes/9.4/limp	7 11.79	Grade II skin	None	None	No	Early puberty (GnRHa-9 years post-HSCT) Short stature Surgery for SUFE (× 4)	83.7 96.9	WB:55% T:100% M:37% B:83%	WB:61% T:94% M:50% 12.7
P18	Synostosis of the proximal radioulnar joint	No/6.59/none	6.59	None	None	Yes > 12 months Pre-HSCT	No	Hypothyroidism Gonadal dysfunction	53.9	WB:100%	WB:100% 9.9
P19	Bilateral SUFE AVN and sclerosis of the left femoral epiphysis	Yes/9.17/limp	9.6	Grade I skin	None	Yes 3 weeks	No	None Surgery for both	30.5	WB:39% T:96% M:37% B:18%	WB:48% T:95% M:39% B:22% 11
P20	AVN of both femoral heads	No/not recorded/abnormal gait	8.5	None	None	Yes > 24 months	No	Thyroiditis after AVN	66.7	WB:99% T:100% M:97% B:100%	WB:100% T:100% M:100% B:100% 11.5
P21	Exostosis of right 5th finger Right ankle exostosis	No/NA/no No/NA/no	7.34 9.95	None	None	Yes 9 months	No		77.5	WB:100%	WB:100% 10.24
P22	Bilateral SUFE Spondyloepimetaphyseal dysplasia/hip dysplasia	Yes/5.47/limp	5.47	None	None	None	GH (post)	Short stature (GH after BD) Hypothyroidism (after BD) Surgical intervention	64.2	WB:100%	WB:100% 8.4
P23	AVN of both hips	Yes/2.4/difficult mobilization	2.4	Grade II skin	None	Yes 18 months Pre-HSCT	Yes (post)	Gonadal dysfunction Short stature Surgical intervention	98.9	WB:100%	WB:100% 8.4
P24	AVN of the left femoral head	No/incidental finding/no	2.38	Grade III gut-steroid refractory	Chronic extensive (gut) 19	Yes > 24 months	No	Hypothyroidism (1 months pre-BD)	94.8	WB:100%	WB:100% 4.9
P25	Osteochondroma of right humerus	Yes/11.7/no	11.7	None	None	None	No	Hemithyroidectomy- thyroid nodule(post)	99.8	WB:73% T:100% M:81% B:68%	WB:73% T:100% M:81% B:68% 13
P26	9th rib exostosis	No/NA/no	4.6	Skin grade II 1.2 months	None	Yes 10 months Pre-HSCT	No	None	8.89	WB:100%	WB:100% 4.57
P27 (previously described by Botto et al.)	Spondyloepimetaphyseal dysplasia/hip dysplasia Bilateral genu valgum deformity	Yes/1.85/abnormal gait	3.6	Grade III skin	Chronic extensive-skin/ITP	Yes > 24 months	GH (post)	Short stature (GH 8 months post-BD)	57.1	WB:100%	WB:100% 10.8
P28	Spondospondyloepimetaphyseal dysplasia	No/6.7/No	6.7	Skin/gut GvHD	Chronic gut GvHD	Yes > 24 months	No	Short stature	75.1	WB:85% T:100% M:85%	WB:90% T:100% M:96% 7.46
P29	AVN of both femoral heads Spondospondyloepimetaphyseal dysplasia of hip/hip dysplasia	Yes/10.9/abnormal gait	11	Skin/gut GvHD	None	Yes > 24 months	No	Short stature	99.6	WB:100%	WB:100% 11.5
P30	Bilateral genu valgum (right > left)	Yes/4/topples while walking/knock knees	4	Grade II Skin GVHD	None	Yes > 24 months	No	Short stature	55.7	WB:22% T:100% M:0	WB: 27% T: 100% M: 13% 7.3
P31	Osteochondroma of right tibia	No	11	Grade II Skin GVHD	None	Yes 17 months	No	Under evaluation for Gonadal dysfunction	97.7	T:100% M:0% B:49%	WB:16% T:100% M:0% B:53% 12.5
P32	Osteochondroma of the right ankle	No	12	Grade II Skin/gut GVHD	None	Yes 12 months	No	None	62.06	WB:74% T: 100% M: 70% B: 90%	WB:72% T: 100% M: 64% B: 91% 14

*aGVHD* acute graft vs host disease, *AI* autoimmunity, *AVN* avascular necrosis of hip, *B* B cell lineage, *BD* bone disease, *cGVHD* chronic graft vs host disease, *GH* growth hormone therapy, *GnRHa* gonadotropin-releasing hormone analogues, *HSCT* hematopoietic stem cell transplant, *HRT* hormone replacement therapy, *M* myeloid lineage, *MTP* metatarsophalangeal joint, *NA* not applicable, *SUFE* slipped upper femoral epiphysis, *T* T cell lineage, *WB* whole blood

### Bone Tumors

While one patient developed a malignant bone disease (P16), 10 patients developed benign bone tumors.

### Malignant Bone Tumor: Osteosarcoma

P16 developed osteosarcoma 13 years post-HSCT. Initially, he presented at the age of 6 years with history of recurrent chest infections and extensive molluscum contagiosum since the age of 5 months. Extensive functional immunological investigations and whole exome sequencing did not identify a defined IEI. At the age of 14, he developed diffuse large B cell lymphoma of the left femur treated with chemotherapy and localized radiotherapy. He underwent successful TCR αβ/CD19 depleted haploidentical transplant (from the father) with T cell add-back at the age of 15 years.

At the age of 17, he developed non-traumatic fractures of the left femur and humerus. There was a family history of repeated fractures in his younger brother. Genetic analysis revealed a homozygous mutation in *COL1A1* c.2167 G > A p(Ala723Thr) in both the patient and younger brother with carrier status in both parents.

At the age of 18 years, he developed a high-grade osteosarcoma in the distal left femur (the same site of previous lymphoma) requiring above knee amputation. Twelve months later, CT chest confirmed localized pulmonary metastases which were surgically resected. Six months later, he developed disease recurrence at the amputation site requiring further resection. He is now 21 years old and receiving palliative care with metastatic lung lesions. He remains 100% donor engrafted.

### Benign Bone Tumor: Osteochondroma

Ten patients (P5, P7, P13, P14, P15, P21, P25, P26, P31, P32) developed osteochondroma at a median time of 10.6 years post-HSCT (range: 4.6–12 years). Four patients (40%) had a diagnosis of WAS, 5 had a diagnosis of SCID (2 undefined, 2 X-SCID, 1 interleukin-7 receptor (IL7R) defect), and one patient had Griscelli syndrome. Nine patients had Treo-based conditioning. MUD PBSCT was the most common donor source (*n* = 6). Six patients had donor chimerism > 95%. Grade I/II aGvHD was seen in 7 patients, and 2 patients developed cGvHD. Eight patients received steroid therapy; 6 received a prolonged course (> 6 months). Five patients were overweight at time of diagnosis of bone disease (P5, P13, P14, P25, and P31). None received HRT pre-bone pathology. While the majority developed lower limb osteochondroma (*n* = 8), 2 patients had either 5th finger (P21) or rib (P26) osteochondroma. Four patients (P7, P13, P14, P25) had pain as a presenting feature, and P7 also had mobility issues. Only one underwent a surgical intervention (P5).

### Skeletal Dysplasia

Six patients developed skeletal dysplasia. P16 was diagnosed with osteogenesis imperfecta 2 years post-HSCT following repeated fractures as previously mentioned. Five patients (P1, P22, P27, P28, and P29) developed other forms of skeletal dysplasia. Diagnoses included WAS (*n* = 2), X-SCID (*n* = 1), congenital neutrophil defect (*n* = 1), familial hemophagocytic lymphohistiocytosis type 2 (FHLH 2; perforin) (*n* = 1). All patients received Treo-based conditioning, Donor and stem cell sources are presented in Supplementary Table 1. All patients had donor chimerism > 95% except P28 who had mixed chimerism. Four patients developed GvHD and required prolonged steroid therapy (> 6 months). Short stature was observed in all patients. Endocrine evaluation for short stature was unremarkable. Targeted skeletal dysplasia genetics (using whole genome sequencing) in P27 and P28 did not isolate any causative mutations. None had HRT pre-bone pathology. P1 developed pain and abnormal gait 12 years post-HSCT. P22 and P29 had incidental diagnosis of skeletal dysplasia while being investigated for other bone pathology (bilateral SUFE and bilateral AVN; respectively). P27 and P28 developed the same pathology(spondyloepimetaphysealdysplasia) at 3.6 and 6.7 years post-HSCT; respectively. While P28 has no abnormal gait, P27 requires crutches and occasional wheelchair support. Of note, P27 had also genu valgum deformity that might have contributed to his physical impairment.

### Vascular Bone Disease; AVN

Nine patients (28%) developed AVN at a median of 6.1 years (range: 2–15 years) post-HSCT. Underlying diagnoses included WAS in 3 cases and SCID in 2 patients: RAG1 SCID (P3), zeta-chain-associated protein kinase 70 (Zap70) SCID (P19). Other diagnoses included X-linked lymphoproliferative disease (XLP1) (P2), combined immunodeficiency (CID) (P4), Crohn’s disease (P23), and X-chromosome-linked inhibitor of apoptosis (XIAP) deficiency (P24). Most patients received 10/10 MUD (*n* = 8). All patients had high level donor engraftment. Seven (78%) patients had a history of aGvHD, and 3 developed cGVHD. Seven patients (77.7%) required prolonged steroid therapy (> 6 months). Four patients were overweight with BMI > 91%. P3 received testosterone therapy 11 months ahead of AVN. Five patients had associated gonadal failure and delayed puberty. Femoral head was the main site of bone pathology in all cases apart from P2 who developed AVN of the right toe, diagnosed at 15 years post-HSCT. Five patients (55%) presented with bilateral AVN. Two patients have additional bone pathologies; P13 developed osteochondroma of right distal femur and left distal fibula with associated mild-moderate genu valgum deformity — 4 years after the diagnosis of AVN. P19 had bilateral SUFE detected concurrently with the diagnosis of AVN. One-third of the cohort required surgical intervention including hip arthroplasty (P4), osteotomy (P19), and hip replacement surgery (P23), while 6 patients are currently being treated conservatively.

### Evolving Bone Disease

Twelve patients developed other bone pathologies: genu valgum deformity (*n* = 10), scoliosis (*n* = 1), and synostosis (*n* = 1). Genu valgum deformity developed at a median time of 8.8 years post-HSCT (range: 3.6–12.6 years). Two-thirds of these patients had underlying diagnosis of WAS (P6, P13, P14) or SCID (P8, P30, P12). Other diagnoses included leucocyte adhesion defect 1 (LAD1) (P11), trico-hepatoenteric syndrome (TTC37) (P10), and FHLH2 (P27). All patients received Treo-based conditioning. P11 received Mel-based conditioning for the second transplant. Majority received a MMUD HSCT (*n* = 7). Stem cell source was PBSC in 4 patients, BM in 3 patients, and cord in 3 patients. Patients (60%) had mixed chimerism at last assessment. Most of the patients developed (7/10) aGvHD, while 2 developed cGvHD. All patients received steroid therapy apart from P17, and 60% of patients (6/10) received steroids for > 6 months. Patients (40%; 4/10) had BMI > 91% at time of onset of bone disease. Interestingly, while 4 patients had associated gonadal failure (P8, P12, P13, P14), P17 entered puberty early ahead of genu valgum deformity. None had HRT/GH therapy prior to development of bone disease apart from P10 who received growth hormone therapy 10 months ahead of the development of genu valgum deformity. Five patients required corrective procedures for management of the genu valgum deformity (P6, P8, P11, P12, P14). P12 required 3 surgeries to correct the deformity. Genu valgum deformity is now fully corrected in 4 out of the 5 patients who had a surgical intervention. P30 and P14 are awaiting surgical procedures.

Other evolving bone disease included scoliosis (P5) and proximal radio-ulnar synostosis (P18). P5 received 2 HSCT (Bu-based myeloablative 9/10 cord HSCT followed by Flu/Mel 9/10 mMUD PBSC HSCT for management of Griscelli Syndrome (2 months after first HSCT)). He developed aGvHD and required steroids for 3 weeks only. He had evidence of gonadal failure and was overweight. He was initially diagnosed with a left fibular osteochondroma 11 years post-HSCT then presented with back pain. Radiology confirmed a diagnosis of scoliosis 12.5 years after HSCT, which has not required corrective surgery. P18 received Bu/Flu MUD BM for CGD. She did not develop GvHD, but she had prolonged steroid use (> 12 months) pre-HSCT. She had associated hypothyroidism and gonadal failure. She presented with inability to rotate her left forearm 6.6 years post-HSCT. Radiology confirmed proximal radio-ulnar synostosis. Both patients had 100% donor engraftment at last follow-up (14 and 10 years, respectively).

### SUFE

Six patients (18.75%) had SUFE at a median of 8.5 years post-HSCT (range: 5.4–11 years). Underlying diagnoses included SCID (*n* = 4; P9, P15, P17, P19) and WAS (*n* = 2; P6, P22). All six patients received Treo-based conditioning with 4 MUD and 2 MMUD HSCT and BM being the stem cell source for 4/6 patients. P15 received DLI for slipping chimerism. Patients (50%; 3/6) had aGvHD. Three patients received steroid therapy, but P15 had a prolonged course of > 6 months for management of extensive chronic GvHD with nephrotic syndrome (5 years of steroid therapy). Interestingly, all patients had normal BMI apart from P9 who was overweight at time of diagnosis of bone disease. P17 received GnRHa for early puberty, and P22 received GH therapy for short stature. All patients developed SUFE at an age ≤ 10 years and 83% (5/6) presented with bilateral SUFE. All patients required a surgical procedure.

## Discussion

Here, we describe the largest cohort of patients with IEI and non-osteopenic bone pathology post-HSCT with follow-up extending to 22 years. Regular follow-up of these patients has demonstrated that non-osteopenic bone pathology is not rare and is present in 9.4% of our cohort of patients. Mean time to develop non-osteopenic bone pathology was 7.8 years with one-third of our cohort developing more than one bone pathology at different time points and more than half diagnosed with bilateral disease. Although the majority of patients (81%) received steroids peri-HSCT with 66% patients (21/32) receiving prolonged steroids for > 6 months (cumulative pre- and post-HSCT steroid exposure), non-osteopenic bone pathology presented years after steroid exposure, and a fifth of patients developed non-osteopenic bone pathology despite no steroid exposure. Pain was the main presenting feature (69% of patients), and 43% of patients required surgical intervention at the time of analysis, with more awaiting procedures. Chronic pain and the need for (repeat) orthopedic surgical interventions have a huge impact on quality of life of a growing child [16].

Specific bone pathology such as the development of osteochondroma has been associated with the use of radiotherapy in malignancies [11]. None of our 10 children who developed osteochondroma underwent radiation as part of their treatment or conditioning regimen, pointing to other factors that might have contributed to the development of bone tumors. Some of the risk factors previously identified in the malignant HSCT setting [12, 14] were present in our cohort, such as age at HSCT < 3 years (100% of our osteochondroma cohort respectively) and male gender (90%), whereas other reported risk factors such as use of growth hormone replacement therapy, or busulfan-based conditioning regime were not found in our osteochondroma cohort. We reported 3% AVN in our cohort, with more than half of patients developing bilateral AVN. Previous groups have reported a similar occurrence rate between 2 and 15% of patients post-HSCT depending on donor choice (increased incidence in MUD HSCT) and increased/prolonged exposure to steroids, ciclosporin, and mycophenolate mofetil [6, 17–20]. In line with these known risk factors, most patients developing AVN in our cohort had increased steroid exposure and an unrelated donor transplant. Of note, while almost all our patients had received a prolonged course of steroids of > 6 months before developing AVN, 2 patients (P13, P19) developed AVN after only receiving a short steroid course (≤ 1 month). Active surveillance is needed even in patients with limited exposure to steroids. Genu valgum deformity was seen among 3% of our patients and was associated with restricted mobility. Moreover, 40% of our patients have required surgical intervention. As previously reported [21], we noted a possible association between genu valgum deformity and obesity with 40% of patients overweight. Early lifestyle intervention for patients with increasing BMI is required to limit the risk for diabetes, hypertension [22], and bone pathology. Although being overweight is a known risk factor for SUFE [23], interestingly, none of the patients with SUFE in our cohort were overweight at time of diagnosis apart from one and all of our patients developed SUFE at a younger age (≤ 10 years) compared to previous reports that SUFE is an adolescent disease associated with obesity [23, 24]. All patients with SUFE in our cohort required surgical intervention with the majority (83%) having bilateral SUFE.

We identified 6 patients with skeletal dysplasia post-HSCT, 5 of which had either diaphyseal dysplasia (*n* = 1) or similar radiographical finding of spondyloepimetaphyseal dysplasia as described by Botto et al. All had stunted growth, and all underwent HSCT < 2 years of age. In contrast to their findings, we did not identify a specific predisposition for skeletal dysplasia in HLH patients post-HSCT. Risk factors for the development of skeletal dysplasia post-HSCT in IEI could include specific exposure to peri-HSCT chemotherapy at an early age (including cumulative steroid exposure pre and post) impacting on bone development and growth. We would recommend that all children presenting with stunted growth post-HSCT to undergo radiological skeletal survey to rule out the development of skeletal dysplasia.

Our results showed increased rates of non-osteopenic bone pathologies among patients with WAS and HLH: 23.5% and 18.8% respectively, compared to ≤ 10% overall. Patients with WAS developed multiple forms of bone pathologies including osteochondroma, genu valgum, SUFE, AVN, and skeletal dysplasia. We could not attribute this to (prolonged) steroid usage alone. Other IEI requiring prolonged steroid therapy, such as patients with CGD [25], had low frequency of bone disease (2.3%) despite being the 2nd most frequent indication for HSCT. Other factors could be responsible for WAS patients’ increased propensity to develop bone disease, in particular osteochondroma. WAS protein (WASp) is a key regulator of the actin cytoskeleton in hematopoietic cells [26] and in osteoclasts. The action of WASp is required for the organization of actin which is essential for osteoclast activity and bone resorption as demonstrated in vivo and in vitro studies [27–29]. Interestingly, Caffey disease, another bone disease associated with hyperostosis and massive subperiosteal new bone formation, has been reported among patients with WAS, independent of HSCT [30, 31]. Bone pathology found in WAS patients pre- and post-HSCT might point to a specific WAS-related etiology causing defective osteoclast activity. Aside from the insults of chemotherapy and an intrinsic defect in the osteoclast cytoskeleton, there may be a pathophysiological predisposition of long-term survivors with a genetic defect in the *WAS* gene to have a higher incidence of various non-osteopenic bone disease, and thus, this cohort of patients should be actively followed up for bone abnormalities.

## Conclusion

Non-osteopenic bone pathology in long-term survivors of allo-HSCT for IEI is not rare. Most patients did not present with complaints until at least 5 years post-HSCT highlighting the need for ongoing bone health assessment. We recommend annual structured musculoskeletal examination, with targeted imaging for reported joint/bone pain, abnormalities in gait/movement, bone deformity, or short stature to allow for early detection and management of non-osteopenic bone pathology. Further studies are also required to evaluate osteopenic bone disease among this cohort of patients, better understand the mechanism and risks factors for both non-osteopenic and osteopenic bone pathology in specific IEI context, which would require a large prospective study with international collaborators.

## Supplementary Information

Below is the link to the electronic supplementary material.

Supplementary file1 (DOCX 18 KB)

## Data Availability

Data and material are available.

## Abbreviations

*Allo-HSCT*: Allogenic hematopoietic stem cell transplantation
*APDS*: Activated PI3K delta syndrome
*AVN*: Avascular necrosis
*BMI*: Body mass index
*BM*: Bone marrow
*Bu*: Busulfan
*CID*: Combined immunodeficiency
*CGD*: Chronic granulomatous disease
*cGVHD*: Chronic graft vs host disease
*CD40L*: Cluster of differentiation 40 ligand
*CHH*: Cartilage hair hypoplasia
*DLI*: Donor lymphocyte infusion
*DOCK8*: Dedicator of cytokine 8
*Flu/Mel*: Fludarabine/melphalan
*FHLH 2*: Familial hemophagocytic lymphohistiocytosis type 2
*GOSH*: Great Ormond Street Hospital
*GH*: Growth hormone
*GnRHa*: Gonadotropin-releasing hormone analogues
*GvHD*: Graft versus host disease
*HLH*: Hemophagocytic lymphohistocytosis
*HRT*: Hormonal replacement therapy
*IEI*: Inborn errors of immunity
*IL7R*: Interleukin-7 receptor
*IPEX*: Immune dysregulation, polyendocrinopathy, enteropathy X-linked
*LAD1*: Leucocyte adhesion deficiency type 1
*MUD*: Matched unrelated donor
*MFD*: Matched family donor
*MMUD*: Mismatched unrelated donor
*OKT3*: Human CD3 monoclonal antibody
*RAG1*: Recombination activating gene 1
*PBSCs*: Peripheral blood stem cells
*SCID*: Severe combined immunodeficiency
*SUFE*: Slipped upper femoral epiphysis
*TBI*: Total body irradiation
*Treo*: Treosulfan
*TTC37*: Trico-hepatoenteric syndrome
*UK*: United Kingdom
*WAS*: Wiskott-Aldrich Syndrome
*WASp*: WAS protein
*XIAP*: X-chromosome linked inhibitor of apoptosis
*XLP1*: X-linked lymphoproliferative disease
*ZAP70*: Zeta-chain-associated protein kinase 70
