# Supplementary material for: Non-osteopenic Bone Pathology After Allo-hematopoietic Stem Cell Transplantation in Patients with Inborn Errors of Immunity
Source: J Clin Immunol. 2023 Mar 17;43(5):1019–31. doi: 10.1007/s10875-023-01465-z (PMC10276082; doi:10.1007/s10875-023-01465-z)
Supplement: Supplementary file 1 — Supplementary file1 (DOCX 18 KB) [file 10875_2023_1465_MOESM1_ESM.docx]

**Supplementary table 1: Demographic data of patients with non-osteopenic bone pathology**

| Patient ID | Gender/Age at HSCT (years) | Diagnosis | Conditioning/transplant |
| --- | --- | --- | --- |
| P1 | F/1.7 | Congenital neutrophil disorder | Flu/Mel/Alem  9/10 MMUD  PBSCs |
| P2 | M/12.3 | XLP | Flu/Mel/Alem  9/10 MMUD  PBSCs |
| P3 | M/0.5 | RAG1 SCID | Flu/Mel/Alem  10/10 MUD  BM |
| P4 | F/13.8 | CID with LPD | Flu/Mel/Alem  10/10 MUD  PBSCs |
| P5 | M/1.5  M/1.9 | Griscelli and HLH | First HSCT:  Bu (MAC)/Cyc/rATG  9/10 MMUD  Cord  Second HSCT  Flu/Mel/Alem  9/10 MMUD  PBSCs |
| P6 | M/1.28 | WAS | Treo/Flu/Alem  10/10 MUD  BM |
| P7 | M/2.6 | WAS | Treo/Flu/Alem  10/10 MUD  BM |
| P8 | F/0.39 | Undefined SCID | Treo/Flu/rATG  7/10 MMUD  Cord |
| P9 | M/1.48 | Undefined SCID | Treo/Flu/Alem  9/10 MMUD  BM |
| P10 | M/5.26 | TTC37 Tricho-Hepatic -enteric syndrome | Treo/Flu/Alem  9/10 CD34+ MMUD  PBSCs |
| P11 | F/0.6  F/8.6 | LAD1 | First transplant  Treo/Flu/rATG  7/10 MMUD  Cord  Second transplant  Flu/Mel/Alem  8/10 CD34+/T cell add-back MMUD  PBSCs |
| P12 | M/0.6 | Gamma chain SCID | Treo/Flu/Alem  7/10 MMUD  BM |
| P13 | M/1.6 | WAS | Treo/Flu/Alem  10/10 MUD  PBSCs |
| P14 | M/1.7 | WAS | Treo/Flu/Alem  9/10 MMUD  PBSCs |
| P15 | M/0.8  M/3.6 | Undefined SCID | First transplant  Treo/Flu/Alem  10/10 MUD  BM  DLI |
| P16 | M/14.4 | Undefined CID/NHL  (COL1A1 homozygous) | Treo/Flu/TT/rATG  TCR ab/CD19 dep haplo-PBSCs with  T cell add-back (suicide gene) |
| P17 | F/0.7 | CHH SCID | Treo/Flu/Alem  9/10 MMUD  BM |
| P18 | F/4.82 | CGD | Bu (RIC)/Flu  10/10 MUD  BM |
| P19 | M/0.9 | Zap70 SCID | Treo/Flu/Alem  10/10 MUD  BM |
| P20 | M/1.9 | WAS | Treo/Flu/Alem  10/10 MUD  PBSCs |
| P21 | M/1 | WAS | Treo/Flu/Alem  10/10 MUD  PBSCs |
| P22 | M/1.39 | WAS | Treo/Flu/Alem  10/10 MUD  PBSCs |
| P23 | M/8 | Crohn’s disease | Treo/Flu/Alem  10/10 MUD  PBSCs |
| P24 | M/1 | XIAP/HLH | Treo/Flu/Alem  10/10 MUD  PBSCs |
| P25 | F/0.4 | Undefined SCID | Flu/Mel/Alem  10/10 MUD  BM |
| P26 | M/0.96 | X-SCID | Treo/Flu/Alem  10/10 MUD  PBSCs |
| P27 | F/1.3 (Previously described by Botto et al) | Perforin HLH | Treo/Flu  8/10 MMUD (6/6)  Cord |
| P28 | M/0.8 | Gamma chain SCID | Treo/Flu/Alem  10/10 MUD  PBSCs |
| P29 | M/1.5 | WAS | Treo/Flu/Alem  10/10 MUD  PBSCs |
| P30 | F/ 1.2 | Undefined SCID | Treo/Flu/Alem  10/10 MFD  PBSCs |
| P31 | M/1.6 | IL7R deficiency SCID | Treo/Cyclo  9/10 MMUD  Cord |
| P32 | M/ 0.8 | X-SCID | Treo/Cyclo/OKT3  Haplo  PBSC |

**Abbreviations:** Alem: Alemtuzumab, BM: bone marrow, Bu: Busulphan, Cyclo: cyclophosphamide, CID: Combined Immunodeficiency, CGD: Chronic Granulomatous Disease, CHH: Cartilage Hair hypoplasia, DLI: donor lymphocyte infusion, Flu: Fludarabine, F: female, haplo: haploidentical donor, HLH: Hemophagocytic lymphohistocytosis, IL7R: interleukin-7 receptor, LAD1: Leucocyte Adhesion deficeincy type1, LPD: lymphoproliferative disorder, M: male MAC: Myeloablative conditioning, MMUD: mismatched unrelated donor, MUD: matched unrelated donor, MFD: matched family donor, Mel: Melphalan, rATG: rabbit Antithymocyte globulin, RAG1: recombination activating gene1, RIC: Reduced intensity conditioning, OKT3: Human CD3 monoclonal antibody, PBSCs: peripheral blood stem cells, SCID: Severe combined immunodeficiency, Treo: Treosulfan, WAS: Wiskott Aldrich Syndrome, XIAP: X- chromosome linked inhibitor of apoptosis, XLP1: X-linked lymphoproliferative disease type 1, ZAP: Zeta-chain-associated protein kinase.
